# Lotus (*Nelumbo nucifera*): a multidisciplinary review of its cultural, ecological, and nutraceutical significance

**DOI:** 10.1186/s40643-024-00734-y

**Published:** 2024-01-27

**Authors:** Hang Yang, Simai He, Qi Feng, Zisen Liu, Shibin Xia, Qiaohong Zhou, Zhenbin Wu, Yi Zhang

**Affiliations:** 1 State Key Laboratory of Freshwater Ecology and Biotechnology, Institute of Hydrobiology, Chinese Academy of Sciences, Wuhan, 430072 China; 2 School of Resources and Environmental Engineering, Wuhan University of Technology, Wuhan, 430070 China; 3 School of Environmental Science and Engineering, Jilin Normal University, Siping, 136000 China; 4 University of Chinese Academy of Sciences, Beijing, 100049 China

**Keywords:** *Nelumbo nucifera*, Ecological adaptation, Sustainable utilization, Nutritional value, Medicinal properties

## Abstract

This comprehensive review systematically examines the multifarious aspects of *Nelumbo nucifera*, elucidating its ecological, nutritional, medicinal, and biomimetic significance. Renowned both culturally and scientifically, *Nelumbo nucifera* manifests remarkable adaptability, characterized by its extensive distribution across varied climatic regions, underpinned by its robust rhizome system and prolific reproductive strategies. Ecologically, this species plays a crucial role in aquatic ecosystems, primarily through biofiltration, thereby enhancing habitat biodiversity. The rhizomes and seeds of *Nelumbo nucifera* are nutritionally significant, being rich sources of dietary fiber, essential vitamins, and minerals, and have found extensive culinary applications. From a medicinal perspective, diverse constituents of *Nelumbo nucifera* exhibit therapeutic potential, including anti-inflammatory, antioxidant, and anti-cancer properties. Recent advancements in preservation technology and culinary innovation have further underscored its role in the food industry, highlighting its nutritional versatility. In biomimetics, the unique "lotus effect" is leveraged for the development of self-cleaning materials. Additionally, the transformation of *Nelumbo nucifera* into biochar is being explored for its potential in sustainable environmental practices. This review emphasizes the critical need for targeted conservation strategies to protect *Nelumbo nucifera* against the threats posed by climate change and habitat loss, advocating for its sustainable utilization as a species of significant value.

**Graphical Abstract:**

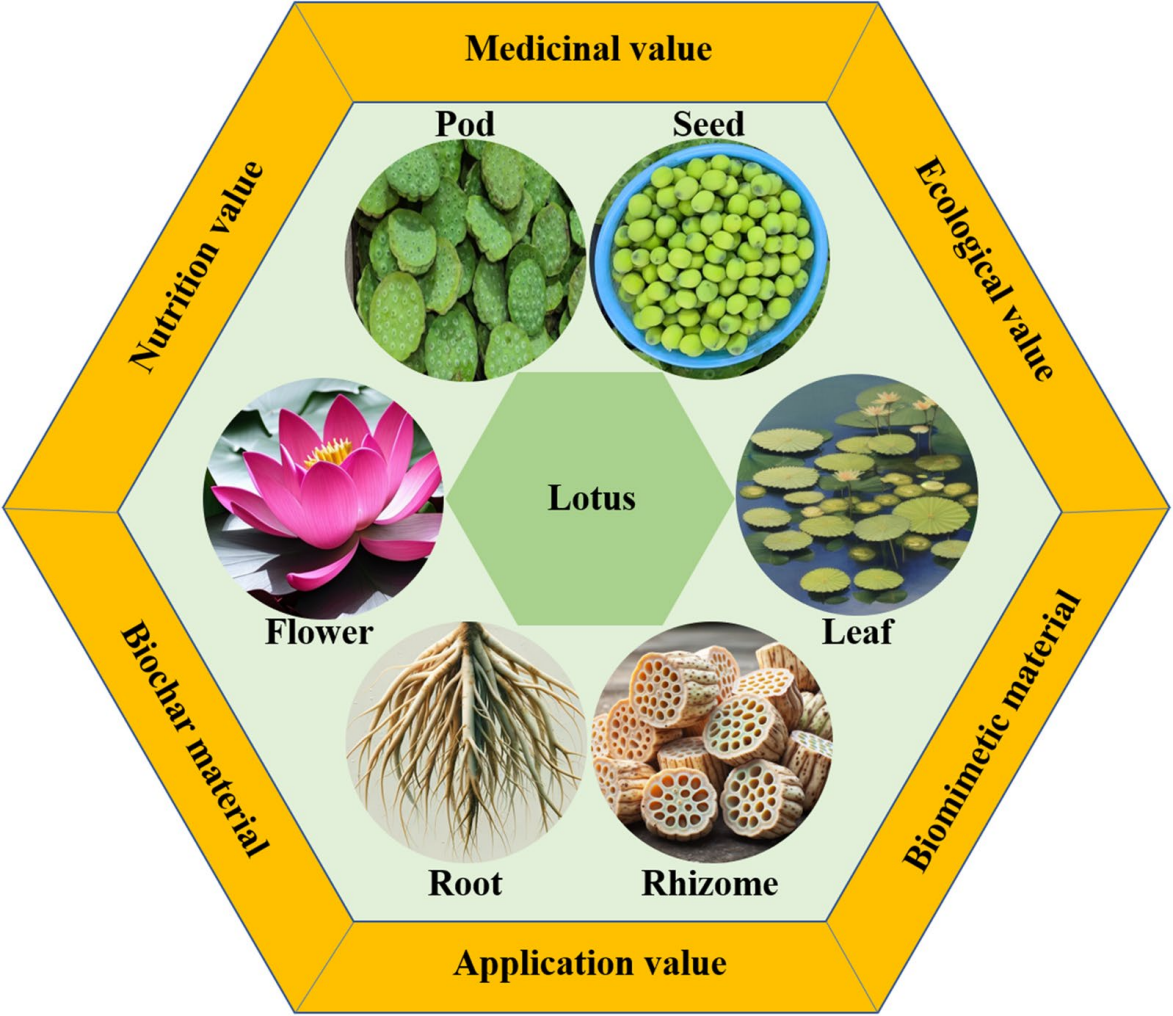

## Introduction

Investigations into aquatic ecosystems, crucial for sustaining biodiversity, have positioned the lotus (*Nelumbo nucifera*) at the forefront of cultural and scientific realms (Zhao et al. 2023). *Nelumbo nucifera* stands out not solely for its historical and aesthetic significance but also for the breadth of research elucidating its complex nature (Salaemae et al. 2018b). Historically, the lotus has been a prominent feature in ancient civilizations, with its seed pod depicted in Egyptian iconography and Vedic texts, highlighting its ceremonial and utilitarian roles (Premathilake and Seneviratne 2015). Beyond its ornamental value, *Nelumbo nucifera* plays a critical ecological role, contributing significantly to the health of aquatic habitats (Jin et al. 2017). The plant has become a focal point in aquatic flora studies, showcasing the broad ecological advantages of comprehensive research (Liu et al. 2020).

Academic research has illuminated various aspects of the lotus, ranging from its phytochemical composition to its structural characteristics (Younis et al. 2023). Investigations into its bioactive elements have revealed substantial medicinal capabilities (Dong et al. 2023; Ibrahim et al. 2024). The notable durability and germination ability of lotus seeds are subjects of intense study, offering potential breakthroughs in seed storage and agricultural methodologies (Salaemae et al. 2018a; Shen-Miller et al. 2013). The superhydrophobic properties of lotus leaves, attributed to their intricate micro and nanostructures, have spurred advancements in biomimetic applications, leading to the development of novel materials and coatings (Srivastav et al. 2021, 2022). Moreover, the buoyant and sturdy architecture of *Nelumbo nucifera* has provided valuable insights into flotation and erosion control technologies (Guo and Liu 2007).

Despite the extensive research on *Nelumbo nucifera*, a noticeable gap persists in the integration of findings across diverse scientific disciplines. Existing studies have often concentrated on isolated aspects of the lotus, such as its phytochemical properties (Mukherjee et al. 2009), ecological roles (Vogel and Hadacek 2004), or potential in biomimicry (Speck and Speck 2021), without a holistic perspective that interconnects these areas. This fragmented approach has led to a compartmentalized understanding, limiting the exploration of *Nelumbo nucifera*'s full potential. There is a notable absence of comprehensive research that amalgamates insights from different scientific fields to provide a cohesive view of the lotus's capabilities and applications. Such an integrative approach is essential for a more profound appreciation and systematic exploitation of *Nelumbo nucifera*'s multifunctional attributes, especially considering its potential impact on health, environmental sustainability, and technological advancement.

This review aims to synthesize existing knowledge on *Nelumbo nucifera*, providing a comprehensive overview of its ecological roles, nutritional value, medicinal benefits, and biomimetic applications. This review adopts a systematic approach, collating data from a wide range of scientific databases and publications. By offering a unified narrative that encapsulates the multifaceted aspects of the lotus, this study seeks to highlight its potential in addressing contemporary challenges in health, environmental sustainability, and technological innovation. Additionally, this review underscores the need for targeted conservation strategies to safeguard this valuable species against the backdrop of global environmental changes. Through this integrative exploration, the review contributes significantly to the understanding of *Nelumbo nucifera*, fostering further research and innovation in various scientific domains.

## Methodology

### Research methodology and literature curation

#### Integrating international databases and keyword strategy

In the execution of this research, a meticulous bibliometric methodology was employed, leveraging renowned databases such as "Science Direct", "Scopus", "PubMed", and "Web of Science". These repositories were instrumental in curating a comprehensive array of scholarly articles and monographs, vital for an all-encompassing review. The investigation was guided by a rigorously formulated keyword strategy, centering on terms crucial to the thematic scope of the study. Keywords, such as "*Nelumbo nucifera*", "Lotus", "Nutrition", "Culture", "Conservation", "Habitat", "Origin", "Distribution", "Taxonomy", "Gene", "Therapy", "Rhizome", "Seed", "Pod", "Food", "Lotus leaf", "Aesthetic", "Biochar", "Biomimicry", and "Lotus effect" were methodically selected and utilized in various syntactical combinations. This approach ensured a thorough and systematic coverage of the research landscape, encompassing diverse disciplines and perspectives pertinent to the multifaceted study of *Nelumbo nucifera*.

#### Rigorous refinement of bibliographic data

Following the initial retrieval, bibliographic data underwent meticulous content analysis. This phase involved the exclusion of sources not directly pertinent to the defined research objectives, such as studies predominantly focused on horticultural, literary and artistic aspects of *Nelumbo nucifera*. The selection predominantly focused on literature detailing the plant's characteristics, growth environments, medicinal values, nutritional components, and practical applications of *Nelumbo nucifera*. This selective process yielded a distilled collection of scholarly works comprising 162 journal articles, each intricately aligned with the aims of the study. This refined compilation formed the foundational corpus for the comprehensive review.

### Research analysis and data sources

#### Bibliometric analysis methodology

The bibliometric analysis methodology employed in this study involved a systematic search on the Web of Science Core Collection database, utilizing "*Nelumbo nucifera*" as the primary keyword. This approach facilitated the analysis of published literature across various timeframes, specifically from 2003 to 2022, and enabled the assessment of publication frequencies by different countries and regions. To visually represent this data, a Python-based visualization of the world map was implemented, graphically depicting the global distribution of publications related to *Nelumbo nucifera*. The study utilized ChatGPT-4.0 to generate a word cloud, visually representing the most prominent terms within the collected literature. Additionally, Vosviewer was employed to create a network diagram, effectively mapping the interconnections and thematic clusters within the research landscape of *Nelumbo nucifera*. This comprehensive analytical method provided a nuanced understanding of the evolution and geographic distribution of *Nelumbo nucifera* research.

#### Source of nutritional composition data

The nutritional composition data presented in this study were meticulously sourced from FoodData Central, an authoritative online database (accessible at https://fdc.nal.usda.gov/ndb/search/list). Operated by the U.S. Department of Agriculture, this comprehensive resource offers detailed and scientifically validated information on the nutritional content of various foods (Fukagawa et al. 2022). The utilization of this database ensured the accuracy and reliability of the nutritional data cited in the review.

### Structural overview of the review

**Fig. 1:**
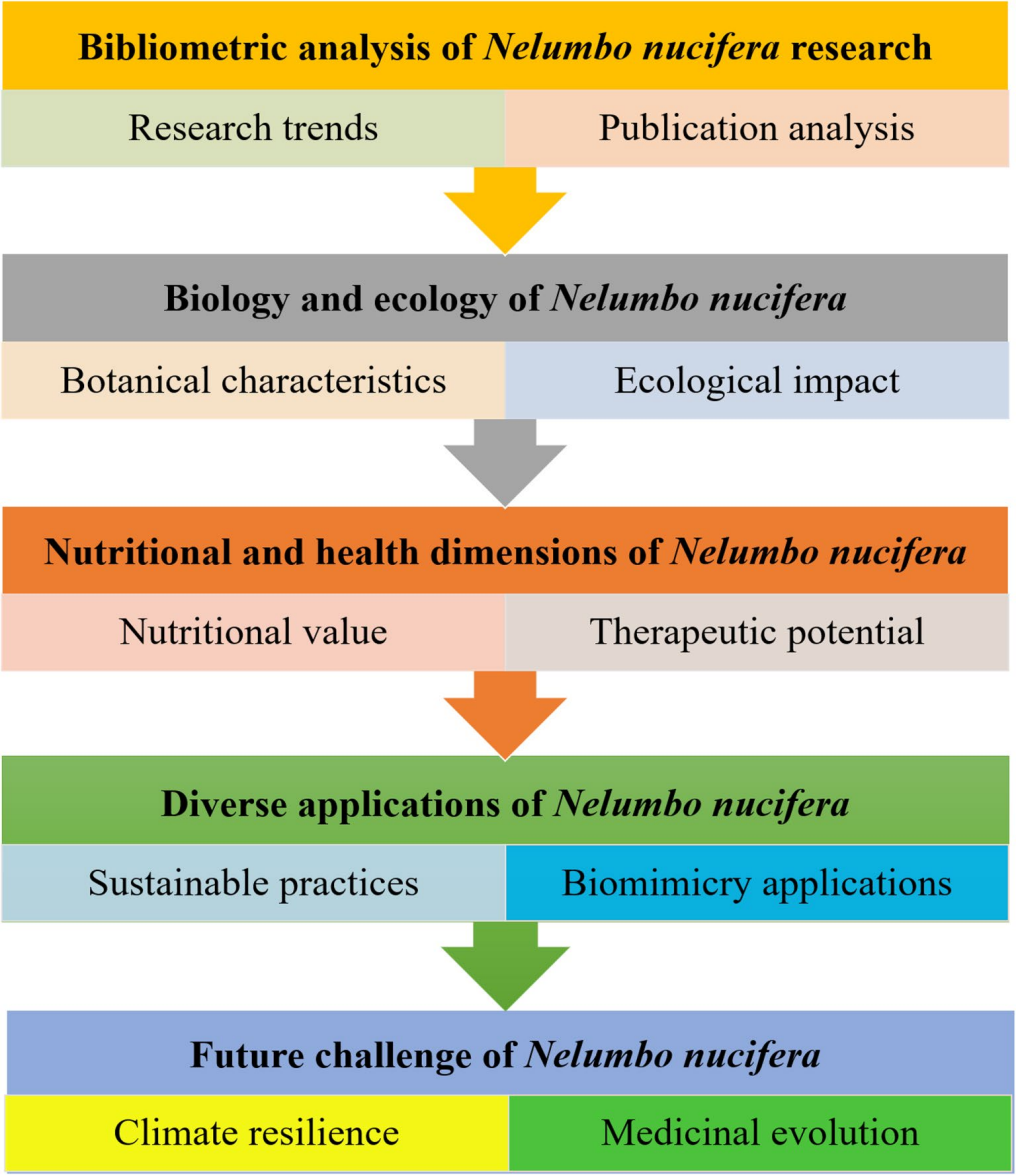
Conceptual framework diagram of this review

This review unfolds in a structured manner, encapsulating the breadth and depth of *Nelumbo nucifera*'s impact across multiple disciplines. Chapter 3 presents a bibliometric analysis, illustrating the growing academic interest in *Nelumbo nucifera* research over two decades. Chapter 4 explores the plant's biology and ecology, detailing its characteristics, habitat, and ecological roles. Chapter 5 focuses on the nutritional and health aspects of the *Nelumbo nucifera*, discussing its dietary contributions and medicinal properties. Chapter 6 highlights *Nelumbo nucifera*'s diverse applications, ranging from environmental purification to its role in biomimicry and sustainable practices. Finally, Chapter 7 evaluates *Nelumbo nucifera*'s potential responses to climate change, medical advancements, and economic impacts, projecting future research directions and applications. Figure 1 displays the conceptual framework diagram of this review.

## Bibliometric analysis of *Nelumbo nucifera* research

**Fig. 2:**
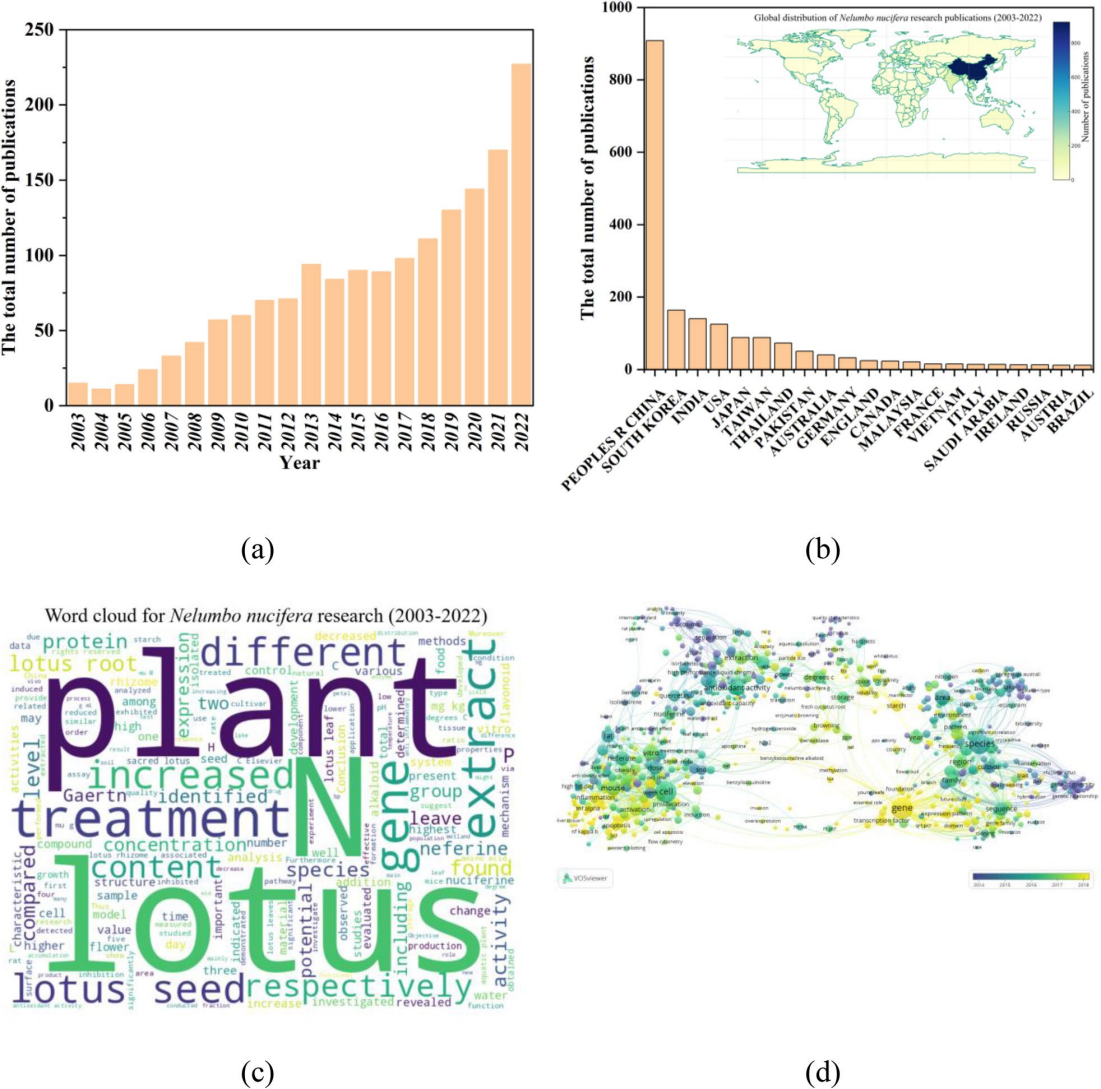
Statistic chart based on Web of Science Core Collection by searching the keywords ‘*Nelumbo nucifera*’: **a** numbers of published articles from 2003 to 2022; **b** numbers of published articles from Top 21 country/region (2003–2022); **c** word cloud; **d** network diagram

The bibliometric analysis of *Nelumbo nucifera* research spanning from 2003 to 2022, illustrated in Fig. 2a, demonstrates a marked escalation in academic interest, reaching its zenith in 2022. This heightened focus is potentially attributable to the growing recognition of the lotus’s ecological and therapeutic properties, likely serving as a catalyst for intensified scientific inquiry into the species. The pronounced peak observed in 2022 may be indicative of critical breakthroughs, increased allocation of research funds, or the organization of specialized symposia dedicated to *Nelumbo nucifera*, thereby augmenting its academic prominence.

Advances in technology, notably in genetic sequencing methodologies and the expansion of bioinformatics, are presumed to have played a significant role in enabling detailed exploration of the lotus (Wu et al. 2022). Moreover, the surge in research activity might also be influenced by the commercial potential of the plant or global phenomena that shape research trajectories (Han et al. 2024). A detailed scrutiny of the publications, funding patterns, and pivotal events during this period is essential for a comprehensive understanding of the factors driving this research trend.

As depicted in Fig. 2b, the geographic distribution of *Nelumbo nucifera* research identifies the People's Republic of China as a preeminent contributor. This dominance is likely reflective of China's longstanding botanical legacy, underscored by the cultural and medicinal relevance of the lotus (Ma et al. 2023), and supported by a substantial scientific framework, possibly bolstered by strategic funding mechanisms (Guo 2009). Other nations such as South Korea, India, and the United States also make significant contributions to the body of research, suggesting their unique scientific or cultural connections to the plant. The limited research activity in many countries may be attributed to the absence of this species in their native flora, underscoring the influence of geographical and ecological factors on the scope and direction of scientific inquiry. An in-depth examination of the thematic content emanating from these different countries and regions would yield valuable insights into the international discourse surrounding *Nelumbo nucifera*.

The word cloud presented in Fig. 2c effectively distills the predominant terminologies employed in the abstracts pertaining to *Nelumbo nucifera* research. Prominent terms such as ‘plant’, ‘lotus’, ‘gene’, ‘extract’, ‘seed’, and ‘treatment’ dominate this visualization, reflecting their frequent occurrence and central importance in studies conducted from 2003 to 2022.

In Fig. 2d, network visualization techniques elucidate the centrality of 'gene' within the research landscape of *Nelumbo nucifera*. This prominence signals an extensive array of genetic research focused on the lotus, highlighting efforts to decode the genetic foundations responsible for its phenotypic characteristics, adaptive resilience, and therapeutic potential. The brightness of recent studies highlights a growing interest in genetic research, likely driven by biotechnological advances such as CRISPR (Pyne et al. 2023) and next-generation sequencing (Li et al. 2023b). These technological innovations have not only facilitated a profound understanding of the lotus genome but also opened avenues for genetic modifications aimed at enhancing its utility across medicinal, ornamental, and ecological domains.

The surge in genetic research related to *Nelumbo nucifera* mirrors wider trends in plant biotechnology (Deng et al. 2023; He et al. 2020), which increasingly emphasize a holistic genomic understanding of plant species. This approach is instrumental in forging pathbreaking solutions to address global challenges such as climate change adaptability, food security, and the advancement of sustainable agricultural practices.

## The biology and ecology of lotus

In the comprehensive study of *Nelumbo nucifera*, attention is given to both its biological attributes and ecological roles, emphasizing its importance in natural ecosystems and varied applications in interdisciplinary fields. The exposition delves into the species' distinct features, including its taxonomic classification and diverse morphological traits. Further analysis of its habitat preferences and adaptability to environmental changes highlights the extensive distribution and ecological versatility of the species. The investigation into the genetic diversity within the *Nelumbo* genus is pivotal for conservation efforts. Concurrently, the exploration of the plant's reproductive strategies and ecological functions sheds light on its crucial role in promoting biodiversity and supporting ecosystem services, thereby underlining the significance of *Nelumbo nucifera* in both ecological and applied scientific contexts.

### Characteristics, taxonomy, and morphological diversity

In the aquatic flora domain, *Nelumbo nucifera*, colloquially known as the lotus, is a paragon of evolutionary sophistication and aesthetic splendor. This species, perennial in its growth, showcases distinctive morphological features that fulfill both functional and cultural roles (Koch et al. 2008). Its hallmark botanical trait, the centrally attached petiole on the peltate leaves, bestows the lotus with unique buoyancy, enabling its leaves to occasionally emerge above the water surface, an adaptation enhancing its photosynthetic efficiency (Khrolenko et al. 2019; Wang and Zhao 2019). The leaves' surface, famed for its superhydrophobic properties, demonstrates the "lotus effect," a self-cleaning mechanism crucial in repelling waterborne sediments and pathogens, thereby safeguarding the plant in its aquatic milieu (Chen et al. 2022).

Taxonomically, *Nelumbo* represents the sole genus in the *Nelumbo*naceae family, a lineage distinct from the visually similar but genetically divergent Nymphaeaceae family (Abd Rasid et al. 2019; Li et al. 2014). Molecular phylogenetic advancements have refined its classification, distinguishing two primary species: *Nelumbo nucifera*, integral to Asian cultural heritage (Fu et al. 2021), and *Nelumbo lutea*, indigenous to North America (Islam et al. 2020).

The morphological diversity within the *Nelumbo* genus is noteworthy, with the two primary species, *Nelumbo nucifera* and *Nelumbo lutea*, exhibiting distinct variations (Lu et al. 2023; Salaemae et al. 2018b). These differences are manifested in several aspects, such as the size of the leaves (Yang et al. 2012), the coloration of the petals (Liu et al. 2023c), and the structure of the seed pods (Punia Bangar et al. 2022). Such variations not only highlight the intrinsic diversity within the genus but also underscore its adaptability to different environmental conditions. The morphological diversity of *Nelumbo nucifera* and *Nelumbo lutea* is a subject of ongoing research, contributing to our understanding of plant adaptation and evolution within aquatic ecosystems (Zhou et al. 2014). This diversity is critical for the ecological functioning of the species, as well as for their cultural and ornamental significance across different regions.

Table 1 displays the overview of different lotus species and their distinctive attributes, encapsulating the taxonomic nuances and morphological diversity of this genus. This table provides a comprehensive perspective on the various lotus species, their origins, unique characteristics, and habitats, thereby enriching the understanding of their ecological and cultural significance.

**Table 1:** Overview of different lotus species and their distinctive attributes

Common name	Scientific name	Origin	Characteristics	Habitat
Sacred Lotus	*Nelumbo nucifera*	Asia and Australia	Large, fragrant pink or white flowers; broad, round leaves	Ponds, lakes, and water gardens
American Lotus	*Nelumbo lutea*	North America	Yellow flowers; large, round leaves with a distinctive notch	Slow-moving rivers and lakes
Egyptian White Water-lily	*Nymphaea lotus*	Egypt	White flowers with a yellow center; night-blooming	Still or slow-moving water bodies
Pygmy Water-lily	*Nymphaea tetragona*	Eurasia	Small white or pink flowers; small, round leaves	Cold, still freshwater bodies
Blue Lotus	*Nymphaea caerulea*	Ancient Egypt	Vibrant blue flowers; used in traditional medicine and for ornamental purposes	Freshwater habitats; ornamental ponds

### Habitat, distribution, and environmental adaptations

The *Nelumbo* genus, celebrated in the domain of aquatic flora, predominantly thrives in stagnant or slow-moving freshwater environments, adaptable to a variety of climatic conditions. *Nelumbo nucifera*, or the Asian lotus, is renowned for its ability to flourish in diverse aquatic habitats, ranging from shallow ponds to expansive lakes (Gowthami et al. 2021). This adaptability is attributable to the plant's physiological and structural adaptations, enabling it to survive and thrive in environments with varying water depths and nutrient levels. The unique root system of *Nelumbo nucifera*, capable of extending deep into the aquatic substrate, plays a crucial role in anchoring the plant and accessing nutrients from the sediment (Seo et al. 2010).

*Nelumbo nucifera*'s distribution spans a wide geographical range, with its presence noted from the Indian subcontinent extending to East Asia (Zhang et al. 2019). The plant's dispersal has been significantly influenced by human activities, including its cultivation for ornamental, religious, and culinary purposes. In contrast, *Nelumbo lutea*, the American lotus, is predominantly found in the temperate zones of North America, thriving in freshwater systems that offer optimal growth conditions (Li et al. 2015). The distinct geographical distribution of these species highlights the genus's adaptability to different environmental settings and its integration into diverse cultural landscapes.

*Nelumbo* species exhibit remarkable adaptive capabilities in response to various environmental stressors. Their seeds are known for their extraordinary dormancy and longevity, with the ability to remain viable for decades or even centuries, a trait that contributes significantly to the genus's resilience and evolutionary success (Rehmani et al. 2023; Sano et al. 2015). This characteristic enables the species to endure fluctuating environmental conditions and ensures their continued propagation. The extensive root system of *Nelumbo nucifera* plays a pivotal role in nutrient uptake and storage, adapting to varying nutrient availabilities in different aquatic environments (Pinardi et al. 2018). Additionally, the thermogenic properties of *Nelumbo* flowers facilitate effective cross-pollination, enhancing the plant's genetic diversity and further enabling its adaptation to diverse ecological niches (Lamprecht et al. 1998). These combined traits underscore the ecological versatility of *Nelumbo*, facilitating its widespread distribution and survival across a range of habitats.

### Genetic diversity and the pursuit of conservation

The genetic landscape of the *Nelumbo* genus exhibits a rich tapestry of ancient lineages, reflecting a history marked by enduring adaptability. Phylogenetic studies utilizing molecular markers have traced the genus's resilience across different epochs, revealing its robust capacity to adapt (Yang et al. 2013). Present-day genetic variation within *Nelumbo* populations signifies a complex evolutionary journey, characterized by recurrent adaptations that have facilitated the genus's proliferation in diverse ecological settings (Abraham et al. 2015; Liu et al. 2023a).

The implementation of advanced genetic assays has been instrumental in analyzing population dynamics and uncovering genetic flux within *Nelumbo*. These techniques have enabled a detailed understanding of intraspecific genetic variation, shedding light on evolutionary trajectories and mechanisms of adaptation (Chen et al. 2023). Despite its evolutionary success, the *Nelumbo* genus faces significant existential threats, including habitat encroachment, water pollution, and climate change, largely driven by anthropogenic factors.

In response to these challenges, concerted conservation efforts are being undertaken. Recognizing the vulnerable status of *Nelumbo* species, global conservation bodies have devised strategic plans for their preservation (Cancio et al. 2016). These efforts include protecting natural habitats, establishing ex-situ conservatories, and creating germplasm banks (Mizuno et al. 2013). Such measures are essential to bolster the resilience of the *Nelumbo* genus against escalating environmental threats, ensuring its survival and continuity for future generations.

### Reproduction, adaptation, and ecological contributions

*Nelumbo nucifera* engages in reproductive strategies that are critical for its proliferation and ecological viability. The species' prominent flowers serve a dual role: aesthetically enchanting and vital for attracting pollinators, thus facilitating cross-pollination. The thermogenic property of the lotus flowers, generating heat to attract pollinators, demonstrates an evolutionary sophistication (Ye et al. 2014). Additionally, asexual reproduction through rhizomes is a key strategy for ensuring the species' perpetuation in favorable environments.

Ecologically, the role of *Nelumbo nucifera* extends beyond reproduction. As a natural biofilter in aquatic ecosystems, it plays a crucial role in assimilating excess nutrients and various pollutants, including heavy metals, thereby reducing contamination impacts (Thongtha et al. 2014). The extensive foliage and root systems of the lotus provide shelter and nourishment to a wide range of aquatic life, enhancing habitat biodiversity. Furthermore, *Nelumbo nucifera* is involved in carbon sequestration, a critical process in combating the challenges posed by climate change, due to its rapid growth and significant biomass generation (Shafique et al. 2020). This ecological aspect not only underscores the lotus's contribution to environmental health but also highlights its role in offsetting carbon emissions.

## The nutritional and health dimensions of lotus

**Fig. 3:**
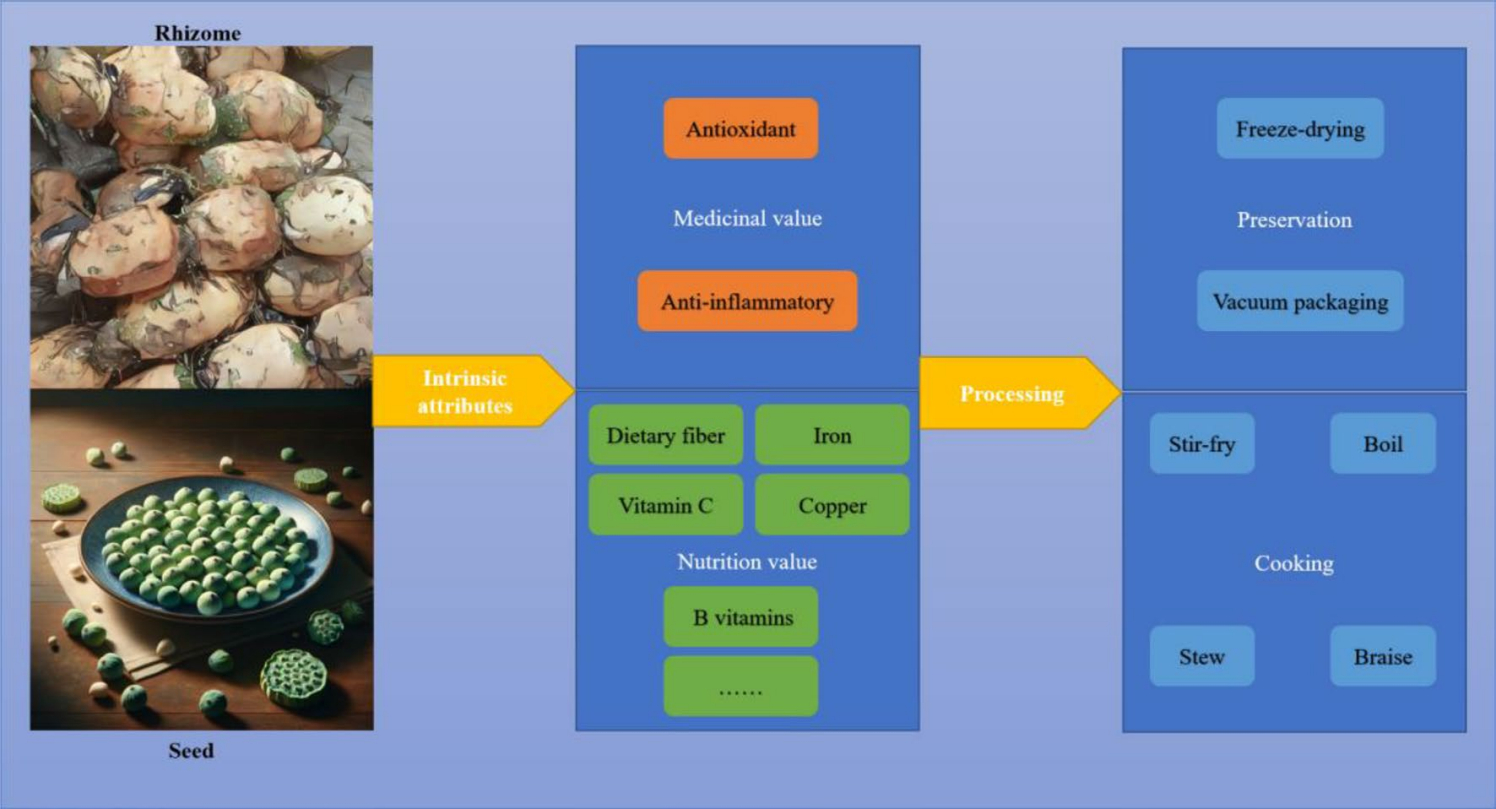
The schematic diagram of the nutritional and health benefits of lotus

This section explores the extensive nutritional profile of *Nelumbo nucifera* and its health implications. Analysis of the lotus’s composition reveals a richness in both macro- and micronutrients, underscoring its dietary importance. Further examination of its therapeutic potentials, in conjunction with health considerations, highlights its significant role in both traditional and modern medicine. The focus then shifts to post-harvest treatments, essential in maintaining the nutritional integrity of *Nelumbo nucifera*. These treatments play a pivotal role in ensuring that the beneficial properties of the lotus are retained from harvest to consumption. Lastly, the section delves into the innovative use of *Nelumbo nucifera* in the culinary world, illustrating its versatility and growing prominence in contemporary gastronomy. Figure 3 presents a schematic diagram depicting the nutritional and health benefits of *Nelumbo nucifera*, providing a visual summary of its multifaceted contributions to nutrition and health.

### The nutritional profile and dietary contributions of lotus

The nutritional value of *Nelumbo nucifera* is primarily anchored in its rhizomes and seeds, each contributing unique dietary benefits. Characterized by a high dietary fiber content, the rhizome plays a crucial role in supporting digestive function and promoting gastrointestinal health (Chen et al. 2020; Li et al. 2020b). It is a rich source of vitamin C, acting as an antioxidant to mitigate oxidative stress while enhancing skin integrity and immune function (Qiu & Chin 2022a). Essential minerals such as iron and copper, integral to hematopoiesis and circulatory health, are also present (Huang et al. 2022b). Moreover, the rhizome's nutrient composition imparts anti-inflammatory properties, recognized for therapeutic benefits in traditional medicine (Chen et al. 2019a).

Lotus seeds are distinguished by their complex carbohydrate structure, facilitating a sustained energy release, beneficial for metabolic equilibrium (Punia Bangar et al. 2022). They provide a complete protein source, encompassing all essential amino acids, a rarity in plant-based proteins (Zhang et al. 2015). The predominantly unsaturated fats in the seeds are conducive to cardiovascular health (Shahzad et al. 2021). Additionally, they contain a spectrum of vitamins, notably B-complex, and essential minerals like magnesium, phosphorus, and potassium, vital for neurological function and metabolic processes (Benammar et al. 2010).

In culinary realms, the lotus rhizome exhibits versatility, being integral to various dishes from stir-fries to snacks, reflecting its gastronomic adaptability. Lotus seeds, traditionally used in ceremonial dishes, now enhance the nutritional and flavor profiles of everyday foods, including soups, desserts, and savory preparations (Yang et al. 2023). Their subtle, nutty flavor and health-promoting qualities position them as a preferred ingredient in contemporary cuisine (Chen et al. 2021a). Collectively, the nutritional properties of *Nelumbo nucifera* rhizomes and seeds, combined with their culinary versatility, underscore their importance in a balanced diet. Table 2 indicates the nutritional profile and bioactive compounds in various parts of *Nelumbo nucifera*, providing detailed insight into their specific nutrient content.

**Table 2:** Nutritional profile and bioactive compounds in various parts of *Nelumbo nucifera*

Part of plant	Water (g/100 g)	Calories (kcal/100 g)	Protein (g/100 g)	Dietary fiber (g/100 g)	Fat (g/100 g)	Carbohydrates (g/100 g)	Sodium (mg/100 g)	Vitamins & minerals	Notable compounds
Lotus rhizome, raw	79.1	311	2.6	4.9	0.1	17.2	40	Rich in B vitamins, vitamin C, potassium, magnesium	Flavonoids, alkaloids
Lotus rhizome, cooked, boiled, drained, with salt	81.4	66	1.58	3.1	0.07	16	281	Rich in B vitamins, potassium, magnesium, high in vitamin C, iron	Flavonoids, alkaloids
Lotus rhizome, cooked, boiled, drained, without salt	81.4	66	1.58	3.1	0.07	16	45	Rich in B vitamins, vitamin C, iron, potassium, magnesium	Flavonoids, polyphenols
Seeds, lotus seeds, raw	77	89	4.13	/	0.53	17.3	1	Contains iron, calcium	Ascorbic acid, calcium oxalate
Seeds, lotus seeds, dried	14.2	332	15.4	/	1.97	64.5	5	Rich in calcium, vitamin C	Flavonoids, quercetin

### Therapeutic potentials and health safeguards

The various components of *Nelumbo nucifera* play a pivotal role in both traditional and contemporary therapeutics (Ming et al. 2013). The rhizome, rich in polyphenols, has garnered increasing recognition for its potent anti-inflammatory effects, as documented in recent scientific studies (Qiu & Chin 2022b). Such properties are vital in managing inflammatory conditions, including rheumatoid arthritis and bronchial asthma. Moreover, the mucilage of the rhizome is hypothesized to possess mucoprotective qualities, offering potential relief for irritated mucosal tissues, thereby serving as an adjunct in treating specific respiratory ailments (Ali et al. 2020).

The seeds of *Nelumbo nucifera*, historically valued in various cultural medicinal practices for their sedative properties, contain a mixture of flavonoids and alkaloids. These compounds demonstrate antioxidant and anti-inflammatory activities (Moon et al. 2019), which are beneficial in preventing chronic diseases marked by oxidative stress and inflammation.

Despite the acknowledged therapeutic benefits of lotus seeds and rhizomes, contemporary research recommends cautious consumption due to potential gastrointestinal disturbances and interactions with pharmaceuticals. The occurrence of allergic reactions, albeit rare, to lotus components also necessitates a gradual integration into diets, especially for individuals with existing food allergies (Andjelković et al. 2017). This cautionary approach underscores the need for balanced utilization of *Nelumbo nucifera* in therapeutic applications, considering both its medicinal potential and possible contraindications.

Expanding the focus beyond seeds and rhizomes, research has delved into the medicinal potential of *Nelumbo nucifera* pods. Extracts from these pods are suggested to possess antipyretic and analgesic properties, potentially offering therapeutic benefits in fever reduction and pain relief (Li et al. 2017a). Historically, these extracts have been applied in treating digestive disorders and cardiac symptoms (Tang et al. 2017).

The leaves of *Nelumbo nucifera*, traditionally utilized for their diuretic properties, are currently under investigation for their anti-obesity effects and potential in regulating blood lipid and glucose levels (Ono et al. 2006). Such regulatory effects are crucial in weight management and diabetes control (Marmouzi et al. 2019). Recent studies on *Nelumbo nucifera* leaf extract have revealed its efficacy in inhibiting the growth of *Streptococcus anginosus*, a bacterium implicated in oral diseases (Lee et al. 2019). This finding underscores the extract's capability as a natural anticariogenic agent, showing promise in oral health applications.

In summary, the multifaceted therapeutic applications of *Nelumbo nucifera* attest to its broad spectrum of medicinal properties. Nevertheless, careful consideration of its contraindications and possible adverse effects is essential. It is advisable to seek medical guidance for the safe use of *Nelumbo nucifera*, particularly for individuals with existing health conditions or those undergoing pharmacological treatments. Table 3 indicates the medicinal values of different parts of *Nelumbo nucifera*, providing a comprehensive overview of its diverse therapeutic applications and associated health benefits.

**Table 3:** Medicinal values of different parts of *Nelumbo nucifera*

Part of plant	Traditional uses	Active compounds	Anti-inflammatory properties	Antioxidant properties	Other medicinal benefits	References
Seed	Sedative, astringent, antidiarrheal	Alkaloids, flavonoids	Yes	Yes	Improves heart health, insomnia treatment	Chen et al. (2021b), Jiang et al. (2018), Sugimoto et al. (2008), Yu et al. (2021)
Rhizome	Digestive health, skin conditions	Polyphenols, alkaloids	Yes	Yes	Skin health, gastrointestinal aid	Dhull et al. (2023), Mukherjee et al. (1997), Tsuruta et al. (2011), Zhu et al. (2018)
Leaf	Cooling effect, treatment of sunstroke	Flavonoids, polyphenols, alkaloids	Yes	Yes	Stress relief, anti-anxiety	Liu et al. (2013), Sharma et al. (2016), Song et al. (2019), Wang et al. (2018), Wu et al. (2017), Zhou et al. (2021)
Flower	Heart tonic, treatment of diarrhea	Flavonoids, glycosides	Yes	Yes	Stress relief, menstrual disorders	Kim et al. (2011), Kumarihamy et al. (2015), Liu et al. (2023b), Nakamura et al. (2013)

### From harvest to shelf: preserving the lotus's bounty

Preservation of *Nelumbo nucifera*'s nutritional and medicinal attributes commences immediately post-harvest, with the harvest timing being pivotal for both rhizomes and seeds. Optimal harvesting of rhizomes is essential to maximize nutritional content and achieve the desired texture. Seeds, on the other hand, should be harvested at the peak of maturity, ensuring a fully developed nutritional profile while avoiding excessive hardness. A meticulous cleaning process for both rhizomes and seeds is imperative to eliminate soil and contaminants.

Post-harvest, the rhizomes undergo blanching, a process crucial for enzyme inactivation to prevent quality degradation and to augment coloration, thereby enhancing shelf life and market appeal. The seeds are subjected to a rigorously controlled drying regimen, essential for preserving their valuable nutrients and extending their longevity (Fang et al. 2023). These initial processing steps play a critical role in maintaining the integrity and quality of *Nelumbo nucifera* parts till they reach consumers.

Recent advancements in preservation technology have markedly enhanced the durability and quality of lotus products. Traditional preservation methods like sun-drying and fermentation have been augmented by contemporary techniques such as cryopreservation and vacuum-sealing. These modern methods aim to extend shelf life while maintaining the nutritional and medicinal virtues of the lotus (Min et al. 2021). Such strategies are instrumental in minimizing oxidative damage and preventing microbial spoilage, thereby preserving the lotus’s bounty in its most efficacious form for consumer utilization (Zhang et al. 2022).

### Culinary innovations: lotus in the food industry

In the dynamic realm of the contemporary food industry, the application of *Nelumbo nucifera*, especially its rhizomes and seeds, has undergone significant innovation. The rhizome, acclaimed for its crisp texture and subtle flavor profile, has been diversely incorporated into culinary creations, ranging from fresh salads to crispy snacks, and even as flavor-enhancing elements in beverages (Park and Lee 2020; Zhu 2017). This component, known for its substantial hydration and appealing taste, is increasingly valued for its versatility across various culinary traditions, particularly as a key ingredient in refreshing summer recipes (Sun et al. 2023).

Simultaneously, *Nelumbo nucifera* seeds have transcended their traditional culinary roles. These seeds are now being ingeniously integrated into modern food products, including nutrient-dense bars, breakfast cereals, and innovative beverages like lotus seed smoothies. Their unique gelatinous property upon cooking is leveraged to impart natural viscosity to culinary preparations such as soups and sauces, thus enhancing texture without relying on synthetic additives (Qi and Zhou 2013).

In the context of the burgeoning trend towards plant-based diets, both the seeds and rhizomes of *Nelumbo nucifera* are being recognized as nutritious and eco-friendly ingredients. Collaborative endeavors among the food industry, nutrition experts, and culinary innovators are focused on enriching various food products with lotus-derived components. This initiative aims to meet the increasing consumer demand for food choices that promote health and well-being, positioning lotus-enriched items at the forefront of the health food market (Dhull et al. 2022). This alignment with contemporary dietary preferences underscores the potential of *Nelumbo nucifera* as a key ingredient in future culinary innovations.

## Diverse applications of lotus

**Fig. 4:**
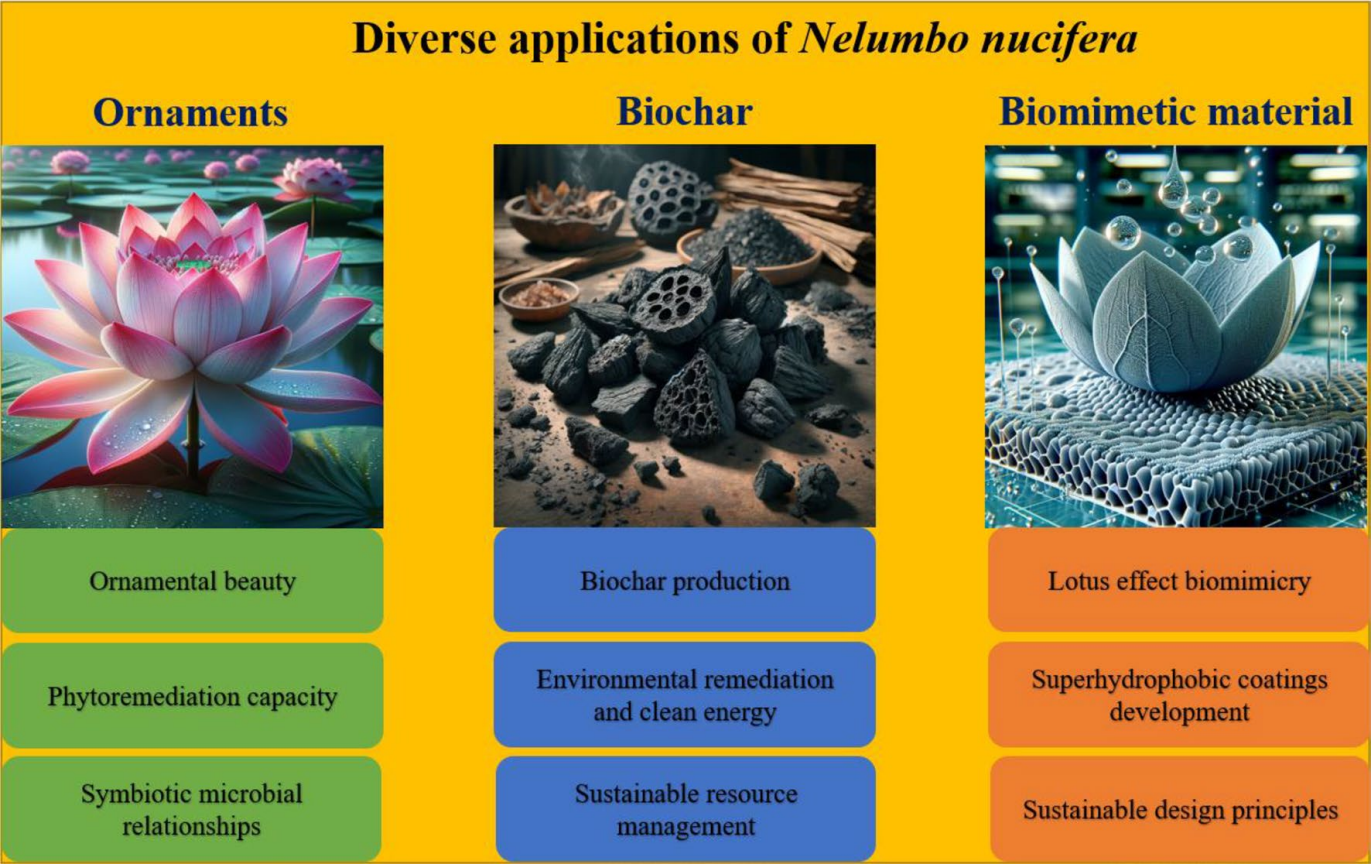
The schematic diagram of the diverse applications of lotus

This section provides a thorough analysis of the varied applications of *Nelumbo nucifera*, extending beyond its aesthetic and nutritional attributes. The initial focus is on the ecological roles of the lotus, particularly its contributions to aesthetic enhancement and water purification. These ecological functions are critical in maintaining healthy aquatic ecosystems and enhancing environmental aesthetics. The discourse then shifts to the plant's role in sustainable practices. This includes the transformation of lotus biomass into biochar, which yield environmental advantages and promote ecological equilibrium. The latter part of this section applies the principles of biomimicry to illustrate how the "lotus effect" is utilized in advanced material design, a testament to the plant's impact on technological advancements. These diverse applications highlight the multifunctionality of *Nelumbo nucifera* and its significant role in various sectors. Figure 4 provides a visual representation of these varied applications, mapping the extensive utility of the lotus across different domains.

### Aesthetic enhancement and ecosystem purification

*Nelumbo nucifera*, renowned for its ornamental beauty, plays a pivotal role in the aesthetic enhancement of aquatic ecosystems (Liu et al. 2022). Landscape designers and horticulturists frequently incorporate this species for its visually striking flowers and lush foliage (Liu et al. 2017). The distinct and elegant appearance of the lotus, with its vibrant blooms and large, floating leaves, significantly elevates the visual appeal of ponds, lakes, and water gardens. Its utilization in landscape design transcends mere aesthetic value, often symbolizing tranquility and purity in various cultural contexts (Lin et al. 2019).

The architectural and design versatility of *Nelumbo nucifera* is further evidenced in its diverse applications across ornamental water features and public gardens (Li et al. 2021). Its capability to adapt to a range of aquatic settings allows for creative and sustainable landscaping solutions. In addition to its visual allure, the rhythmic seasonal flowering and foliage decay of the lotus contribute to dynamic and ever-changing landscapes, offering year-round visual interest and ecological benefits (Sheng et al. 2022).

*Nelumbo nucifera* plays an integral role in water purification within its native aquatic habitats (Abd Rasid et al. 2019). The plant's extensive root system, submerged beneath the water's surface, could efficiently absorb and sequester a variety of aquatic pollutants, including heavy metals and nutrients, thereby contributing significantly to the improvement of water quality (Liu et al. 2016; Yuan et al. 2019). This phytoremediation capacity of *Nelumbo nucifera* is particularly valuable in mitigating eutrophication (Xu et al. 2019), which is often caused by nutrient overload leading to harmful algal blooms and subsequent ecological imbalances (Fan et al. 2018).

In addition to its direct pollution absorption capabilities, the rhizosphere of *Nelumbo nucifera* fosters a thriving ecosystem of microorganisms (Mohd Zaini et al. 2021). These microbial communities engage in symbiotic relationships with the plant, playing a crucial role in the decomposition of organic materials (Chaudhari et al. 2023). This process not only maintains water clarity but also contributes to the overall health of the aquatic ecosystem. The presence of *Nelumbo nucifera*, therefore, extends beyond aesthetic enrichment to encompass vital ecological functions, notably in sustaining the purity and balance of freshwater habitats (Zhang et al. 2021).

### Transforming lotus into biochar: sustainable practices and Outcomes

*Nelumbo nucifera*, symbolizing purity and renewal, extends its ecological contributions through transformation into biochar, a process transcending its aesthetic appeal. This conversion, achieved via controlled pyrolysis—the thermochemical decomposition of organic matter at elevated temperatures in an oxygen-deprived environment—transforms the entire plant, including pods, stems, leaves, and rhizomes, into a carbon-rich substance (Wang et al. 2022). This substance is characterized by a porous structure and extensive surface area, enhancing its applicability in diverse ecological and industrial contexts (Wu et al. 2021). The production of biochar exemplifies sustainable resource utilization, embodying circular economy principles by repurposing plant residues that would otherwise undergo decomposition. This biochar significantly benefits soil health, augmenting fertility and moisture retention, and acts as a carbon sink, thus contributing to climate change mitigation efforts (Rao et al. 2022).

In environmental remediation, the adsorptive properties of lotus-derived biochar are exploited for wastewater treatment, effectively sequestering heavy metals and nutrients and thereby reducing water pollution (Hou et al. 2021; Wu et al. 2018a). This characteristic also paves the way for its inclusion in energy storage and electrochemical devices (Dang et al. 2021; Zhou et al. 2022), potentially enhancing the efficiency and lifespan of batteries (Li et al. 2017b). The application of lotus biochar in these advanced technological areas highlights the plant's multifunctionality, positioning it as a key component in clean energy and environmental purification strategies. Table 4 presents a comprehensive overview of the preparation and applications of lotus-based biochar.

**Table 4:** Preparation and application of lotus-based biochar

Carbon source	Preparation parameter	Application	Efficiency	References
Lotus pod	Carbonization temperature: 800 ºC; Holding time: 2 h; Atmosphere: argon gas	Energy storage	Specific capacitance of 160 F/g in a three-electrode system	Atchudan et al. (2022a)
Lotus pod	Carbonization temperature: 800 ºC; Holding time: 2 h; Atmosphere: argon gas	Clean energy harvesting	Overpotential of 111 mV with the Tafel slope of 69 mV/dec	Atchudan et al. (2022b)
Lotus pod	Carbonization temperature: 600 ºC; Holding time: 2.5 h; Atmosphere: argon gas	Cadmium (II) adsorption	Adsorption capacity of 51.18 mg/g for cadmium (II)	Chen et al. (2018)
Lotus pod	Carbonization temperature: 1200 ºC; Holding time: 2 h; Atmosphere: argon gas	Anode-active material for sodium-ion batteries (SIBs)	An optimized reversible capacity of 328.8 mAh/g	Wu et al. (2019)
Lotus pod	Co-pyrolysis with magnesite; Pyrolysis temperature: 700 ºC; Holding time: 2 h; Atmosphere: nitrogen gas;	Phosphorus adsorption	Adsorption capacity of 523.91 mg/g for phosphorus	Fang et al. (2022)
Lotus pod	Carbonization temperature: 600 ºC; Holding time: 1 h; Atmosphere: argon gas	Electrode material for supercapacitors	Specific capacitance of 165 F/g	Pu et al. (2015)
Lotus pod	Carbonization temperature: 650 ºC; Holding time: 2 h; Atmosphere: nitrogen gas	CO_2_ capture	CO_2_ uptake of 6.20 mmol/g at 0 ºC and a bar	Xie et al. (2022)
Lotus leaf	Carbonization temperature: 1000 ºC; Holding time: 2 h; Atmosphere: argon gas	Interlayer for lithium sulfur batteries	Discharge capacity of 442 mA h/g	Wang et al. (2023)
Lotus leaf	Carbonization temperature: 800 ºC; Holding time: 2 h; Atmosphere: nitrogen gas	Anodes for sodium ion batteries	Discharge capacity of 250 mA h/g	Wang and Su (2021)
Lotus leaf	Carbonization temperature: 800 ºC; Holding time: 2 h; Atmosphere: argon gas	Medium for solar-driven steam generation	Water evaporation rate of 1.30 kg/m^2^ h and a solar-vapor conversion efficiency of 77.5%	Guo et al. (2020)
Lotus pollen	Carbonization temperature: 800 ºC; Holding time: 2 h; Atmosphere: nitrogen gas	Reactive Black 5 adsorption	Adsorption capacity of 615.6 mg/g for Reactive Black 5	Ye et al. (2022)
Lotus stamens	Carbonization temperature: 800 ºC; Holding time: 2 h; Atmosphere: nitrogen gas	Electrode material for supercapacitors	Specific capacitance of 322.8 F/g	Chen et al. (2021c)
Lotus stem	Carbonization temperature: 800 ºC; Holding time: 1 h; Atmosphere: nitrogen gas	CO_2_ capture	CO_2_ uptake of 6.17 mmol/g at 0 ºC and 1 bar	Wu et al. (2018b)

The scaling of lotus biochar production necessitates a balance between its environmental benefits and the energy expended in its creation. A thorough lifecycle assessment is crucial to confirm that the environmental impact is positive and that the production process is in line with sustainable development objectives. Consequently, *Nelumbo nucifera* transitions from a cultural symbol to an agent of ecological innovation, reflecting a commitment to environmental conservation and sustainable resource management amid global challenges.

### Biomimicry: harnessing the lotus effect in material design

Biomimicry, a discipline focused on emulating nature’s designs for technological advancements, finds a prime example in *Nelumbo nucifera*. The lotus leaf’s surface, distinguished by its unique micro and nanotopography, displays superhydrophobic properties, commonly known as the "lotus effect" (Zhou et al. 2023). This phenomenon, wherein water droplets form beads and roll off the leaf, removing surface contaminants, has catalyzed the development of a range of materials and coatings that replicate this self-cleaning capability (Li et al. 2022b). These innovations, designed to repel water and deter the accumulation of dirt and pathogens, are particularly beneficial in industries such as construction, textiles, and medical device manufacturing.

**Fig. 5:**
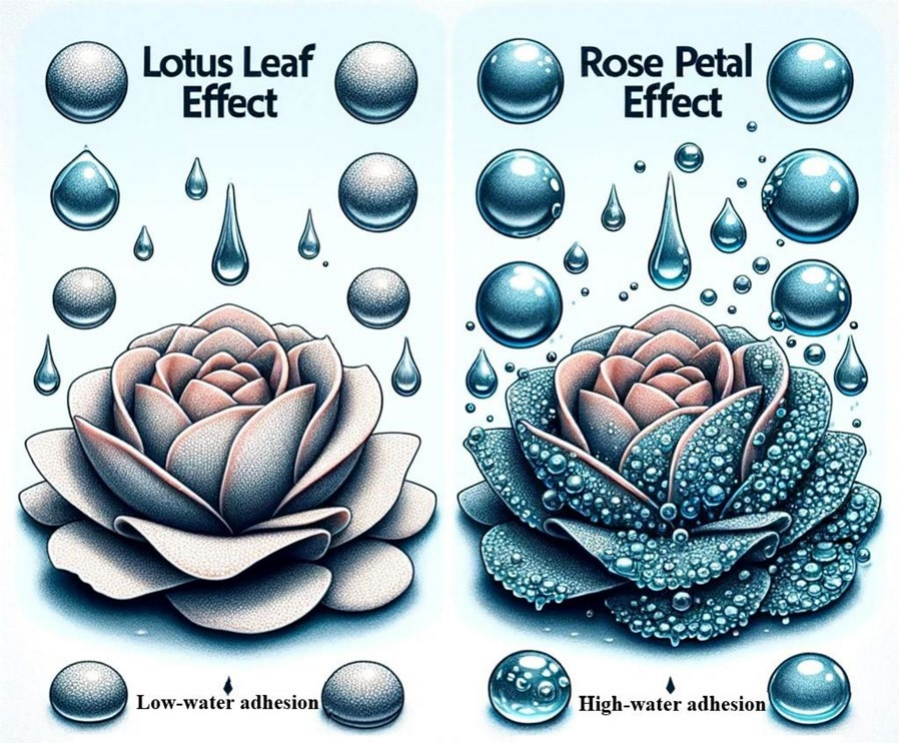
Illustration of rose petal and lotus leaf effects

Recent progress in biomimetic materials science is evident in several research endeavors. For example, one study has pioneered tunable adhesion superhydrophobic coatings (TASCs) that can imitate both the lotus effect and the petal effect by altering spraying pressure during fabrication (Li et al. 2022a). These coatings demonstrate distinctive water droplet behaviors, applicable in areas like droplet transportation and anti-icing. Another research project focused on creating a superhydrophobic coating using ultrahigh molecular weight polyethylene (UHMWPE), modifying quenching temperatures and solvent compositions to achieve surfaces with variable water adhesion properties, ranging from the high-adhesion “rose petal effect” to the low-adhesion “lotus leaf effect” (Sun et al. 2021). Figure 5 visually contrasts the “rose petal effect” with the “lotus leaf effect,” elucidating the differential behaviors of water droplets on these surfaces. These techniques enable the fabrication of multifunctional surfaces suited for diverse applications, including droplet manipulation, oil absorption, and self-cleaning. Collectively, these studies represent significant advancements in surface engineering, drawing inspiration from the innate properties of the *Nelumbo nucifera* leaf.

Beyond their surface properties, the structural adaptations of *Nelumbo nucifera*, especially its stem characterized by air-filled cavities, are being explored for their potential in developing lightweight materials that could play a crucial role in energy absorption and conversion (Shi et al. 2023; Tian et al. 2019). Table 5 presents an exhaustive overview of the methodologies employed in the development of lotus-inspired biomimetic materials, along with their varied applications, highlighting the plant's significant contributions to the forefront of material science.

**Table 5:** Preparation and applications of lotus related biomimetic materials

Inspiration source	Biomimetic materials	Application	Efficiency	References
Lotus leaf	Lotus leaf-like structured gauze (Lotus@Gauze)	Wound dressing	Excellent antiadhesive and antibacterial effects, enhanced infected wound healing compared to clinically available gauzes	Li et al. (2020a)
Lotus leaf	PCL nanofibrous mats modified with HMDSO	Antiadhesive barrier	Enhanced surface hydrophobicity and reduced cell adhesion in vitro	Klicova et al. (2022)
Lotus leaf	PVA/CS composite film spray coated with beeswax and SiO_2_ nanoparticles	Antibacterial adhesive packaging film	Excellent physicochemical properties and anti-bacterial adhesion	Huang et al. (2022a)
Lotus leaf	Superhydrophobic surface on dielectric layer of droplet-based electricity generator	Raindrop energy, harvesting self-cleaning DEG for outdoor use and raindrop acidity alert	High electricity output, wide operational droplet volume range, and excellent performance in raindrop energy harvesting, self-cleaning, and raindrop acidity alert applications	Yoo et al. (2022)
Lotus leaf	Micrometer-sized spherulites	Superhydrophobic coatings	Water contact angles over 150° and water shedding angles below 10° for coatings	Bai et al. (2018)
Lotus rhizome	Kevlar aerogel	Sewage treatment and all-day fresh water production	High catalytic efficiency of 98.91% for pollutant degradation and photothermal conversion efficiency of 81.2%	Li et al. (2023a)
Lotus flower	Manganese dioxide (MnO_2_) microspheres with nanostructures coated on Mg alloy surface and modified with stearic acid	Superhydrophobic self-cleaning coatings	Robust self-cleaning performance in air and oil	Zang et al. (2020)

The translation of *Nelumbo nucifera*'s natural designs into practical applications, however, demands meticulous consideration to preserve ecological balance. Innovations inspired by the lotus require responsible development, ensuring that the biomimetic process does not inadvertently contribute to environmental harm. This exploration of the lotus effect and related phenomena emphasizes the criticality of adhering to sustainable design principles, marrying the ingenuity of nature's designs with human innovation, whilst ensuring the integrity of the natural environment is upheld.

## Charting the future of lotus

As the future unfolds, *Nelumbo nucifera* emerges at the nexus of climatic resilience, medical potential, and socio-economic impact. Investigative efforts into its responses to climate variability shed light on the plant's survival strategies, revealing adaptive mechanisms crucial under changing environmental conditions. Concurrently, the exploration of *Nelumbo nucifera*'s medicinal properties is evolving, bridging traditional uses with contemporary pharmacological advancements. This exploration not only enhances our understanding of its therapeutic capabilities but also opens new avenues for medical applications. In tandem, the commercial and artistic roles of *Nelumbo nucifera* are expanding, illustrating its diverse contributions on a global scale. These aspects collectively highlight the plant's broad relevance and potential for future applications in various fields. As *Nelumbo nucifera* continues to adapt and evolve, its multifaceted significance across ecological, medical, and socio-economic spheres underscores its enduring global importance.

### Climate change resilience: the lotus's response

The resilience of lotus to climate change is garnering increasing attention in aquatic botany (Vo et al. 2021). As the climate crisis intensifies, marked by significant shifts in global weather patterns due to anthropogenic activities, its impact on freshwater ecosystems emerges as a critical research focus (Liu et al. 2015; Park et al. 2023).

However, the long-term resilience of *Nelumbo nucifera* under progressive climate change scenarios remains uncertain. Research has identified potential vulnerabilities, including diminished seed germination rates linked to elevated water temperatures and stunted growth in response to increasing salinity (Liu et al. 2014; Zhou et al. 2012), a consequence of rising sea levels and diminished freshwater influx. Yet, the genetic diversity within lotus populations presents an opportunity to identify strains with enhanced resistance to climatic stressors (Chen et al. 2019b).

The cultivation of these resilient strains, coupled with strategic conservation initiatives, is imperative for the preservation of *Nelumbo nucifera* populations. Although the species' adaptability is apparent, a prudent approach is required to address the challenges posed by extreme climatic events and environmental shifts. A comprehensive understanding that merges the innate resilience of *Nelumbo nucifera* with active human intervention is crucial to ensure its ongoing survival and ecological relevance.

### Lotus in medicine: unlocking therapeutic avenues

The incorporation of *Nelumbo nucifera* into the medical domain represents a synthesis of traditional wisdom and contemporary pharmacological research. Historically, in various cultural medical practices, *Nelumbo nucifera* has been valued for its therapeutic properties (Sharma et al. 2017). Its seeds, known for their calming effects, are utilized in Chinese medicine, while the rhizomes are consumed for their anti-inflammatory and antioxidant properties (Chen et al. 2007; Yang et al. 2022). These longstanding uses highlight the plant's potential as a therapeutic agent, a notion sustained across generations.

Recent scientific inquiries have increasingly sought to validate the medicinal claims associated with *Nelumbo nucifera*. Investigations have identified bioactive constituents, including alkaloids and flavonoids, that exhibit a range of pharmacological activities, such as anti-cancer, anti-diabetic, and neuroprotective effects (Pyne et al. 2023). Furthermore, the potent antimicrobial properties of *Nelumbo nucifera* extracts against specific drug-resistant bacteria have been documented, suggesting their potential role in addressing antibiotic resistance (Matthews and Haas 1993).

The journey from traditional remedies to scientifically verified pharmaceuticals necessitates rigorous research to delineate the therapeutic profiles of *Nelumbo nucifera*-derived compounds. This process entails determining their safety, efficacy, appropriate dosages, and interactions with other medications. Regulatory supervision is paramount to minimize risks associated with the unsupervised use of these bioactive substances, which might otherwise lead to adverse health impacts.

As the field of pharmacology evolves, the integration of *Nelumbo nucifera*-based substances into modern therapeutics appears increasingly feasible. The amalgamation of traditional medicinal knowledge, cutting-edge research, and pharmaceutical industry resources has the potential to foster the development of standardized and effective *Nelumbo nucifera*-based treatments. The future role of *Nelumbo nucifera* in medicine hinges on striking a balance between ancestral insights and stringent scientific verification. Given its extensive array of bioactive compounds, *Nelumbo nucifera* stands poised to make significant contributions to medical therapeutics, contingent on exploration that is both precise and judicious.

### The lotus economy: commercial and artistic implications

*Nelumbo nucifera* transcends its aquatic origins, significantly influencing both economic and artistic domains with its diverse applications. From an economic perspective, it exemplifies sustainable agricultural practices, contributing notably to both local and global markets (Zahoor et al. 2021). Its components, including nutrient-rich rhizomes and viable seeds, are integrated into various food products (Showkat et al. 2021), thereby enriching culinary diversity. This multifaceted utility ensures a consistent market demand, encouraging the cultivation of *Nelumbo nucifera* as an economically and environmentally sustainable crop (La-ongsri et al. 2009).

Artistically, *Nelumbo nucifera* has emerged as a powerful symbol across cultures (van Schaik et al. 2015). Its depiction in art and literature, spanning centuries, encapsulates themes of purity and enlightenment. Artists and writers have consistently been captivated by its emblematic form, incorporating its imagery into a range of creative works that resonate with its intrinsic elegance (Zhang et al. 2023). The demand for *Nelumbo nucifera*-inspired artistic and literary creations further bolsters its economic value, with such items often sought after by collectors and aficionados.

*Nelumbo nucifera* represents an amalgamation of economic and artistic narratives, standing as a beacon of sustainable development and creative inspiration. It generates tangible economic benefits for cultivators and industries while enriching cultural and artistic communities. Looking ahead, the fusion of advanced agricultural methodologies, innovative artistic interpretations, and new market strategies is poised to enhance the role of *Nelumbo nucifera* in the global economy and the realm of arts. Thus, *Nelumbo nucifera* epitomizes the synergy of economic feasibility and cultural resonance, symbolizing an equilibrium between material prosperity and artistic manifestation.

## Conclusion

This review, encompassing a multifaceted analysis of *Nelumbo nucifera*, underscores its profound significance in ecological, nutritional, medicinal, and biomimetic domains. The findings highlight *Nelumbo nucifera*'s adaptability to diverse environmental conditions, reinforcing its ecological importance in aquatic ecosystems through roles such as biofiltration and habitat enhancement. The species' nutritional profile, rich in essential nutrients, and its versatility in culinary applications, demonstrate its contribution to dietary health and gastronomic innovation.

Medicinally, *Nelumbo nucifera* emerges as a repository of bioactive compounds with considerable therapeutic potential, offering promising avenues in the treatment and management of various health conditions. Its integration into traditional and modern medicine underscores its enduring relevance and the necessity of bridging ancient wisdom with contemporary scientific validation. The review also delves into the innovative applications of *Nelumbo nucifera* in biomimicry, particularly the exploitation of the "lotus effect" in material science, highlighting its potential in advancing sustainable technological solutions.

However, the review identifies a critical need for integrated, interdisciplinary research approaches to fully exploit the potential of *Nelumbo nucifera*. While substantial progress has been made, there remains a gap in the holistic understanding of the plant's capabilities, necessitating a concerted effort to amalgamate insights from various scientific disciplines. Such an integrated approach is pivotal for harnessing the full spectrum of *Nelumbo nucifera*'s benefits, addressing global challenges in health, sustainability, and innovation.

Furthermore, the review emphasizes the urgency of conservation strategies to protect *Nelumbo nucifera* against threats posed by climate change, habitat loss, and anthropogenic pressures. The preservation and sustainable utilization of this species are paramount for maintaining its ecological, nutritional, and medicinal values for future generations.

In conclusion, *Nelumbo nucifera* stands as a symbol of ecological resilience, nutritional richness, therapeutic promise, and biomimetic inspiration. Its comprehensive study and sustainable management are imperative, promising significant contributions to diverse fields of science, health, and technology. As the global community faces unprecedented environmental and health challenges, *Nelumbo nucifera* offers a beacon of hope and a testament to the power of integrating traditional knowledge with modern scientific inquiry.

## Data Availability

Data will be made available upon reasonable request.
