# Correction: Lotus (*Nelumbo nucifera*): a multidisciplinary review of its cultural, ecological, and nutraceutical significance

**DOI:** 10.1186/s40643-024-00829-6

**Published:** 2024-12-20

**Authors:** Hang Yang, Simai He, Qi Feng, Zisen Liu, Shibin Xia, Qiaohong Zhou, Zhenbin Wu, Yi Zhang

**Affiliations:** 1 State Key Laboratory of Freshwater Ecology and Biotechnology, Institute of Hydrobiology, Chinese Academy of Sciences, Wuhan, 430072 China; 2 School of Resources and Environmental Engineering, Wuhan University of Technology, Wuhan, 430070 China; 3 School of Environmental Science and Engineering, Jilin Normal University, Siping, 136000 China; 4 University of Chinese Academy of Sciences, Beijing, 100049 China

**Correction: Bioresources and Bioprocessing (2024) 11:18** 10.1186/s40643-024-00734-y

Some of the original images in this paper (Yang et al. 2024) were generated using AI to visually illustrate key concepts; however, it was later discovered that some of the images contained inaccurate information. Additionally, the description of Table 1 was identified as being inaccurate, and we sincerely apologize for any confusion this may have caused. To address this, we have carefully reviewed and replaced each AI-generated image that lacked precision with manually taken, authentic photographs, and revised the description of Table 1 to ensure its accuracy and clarity. These replacement images are free from post-editing and faithfully capture the intended scientific details, ensuring both accuracy and clarity. Our goal is to provide readers with reliable visual content that supports a clear understanding of the material, without raising concerns about misrepresentation or copyright issues. Below is a list of the original images and tables, along with their revised counterparts, which now accurately reflect the content discussed in the paper.

**Incorrect graphical abstract:** 
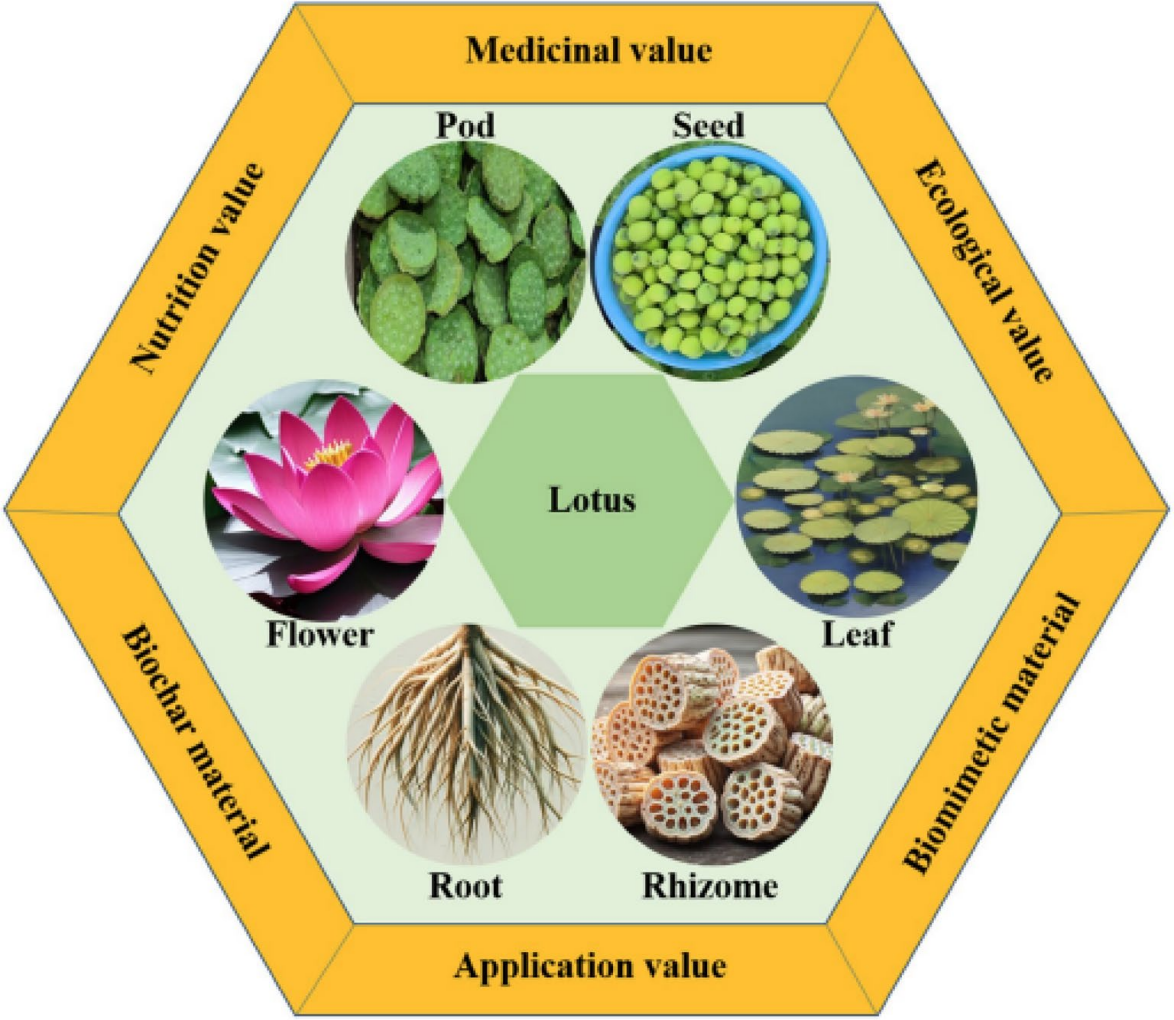


**Corrected graphical abstract:** 
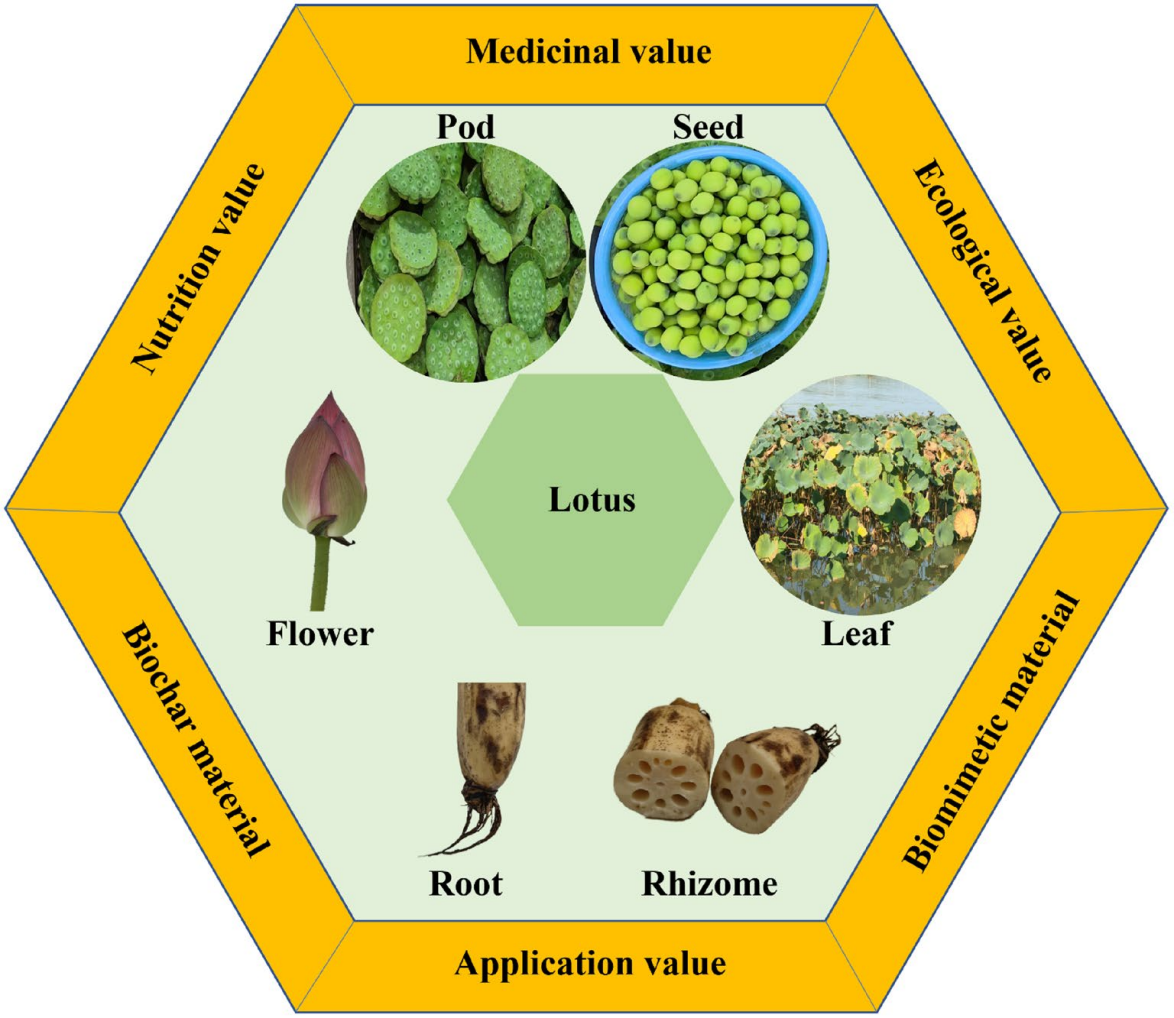


Incorrect Fig. 3 
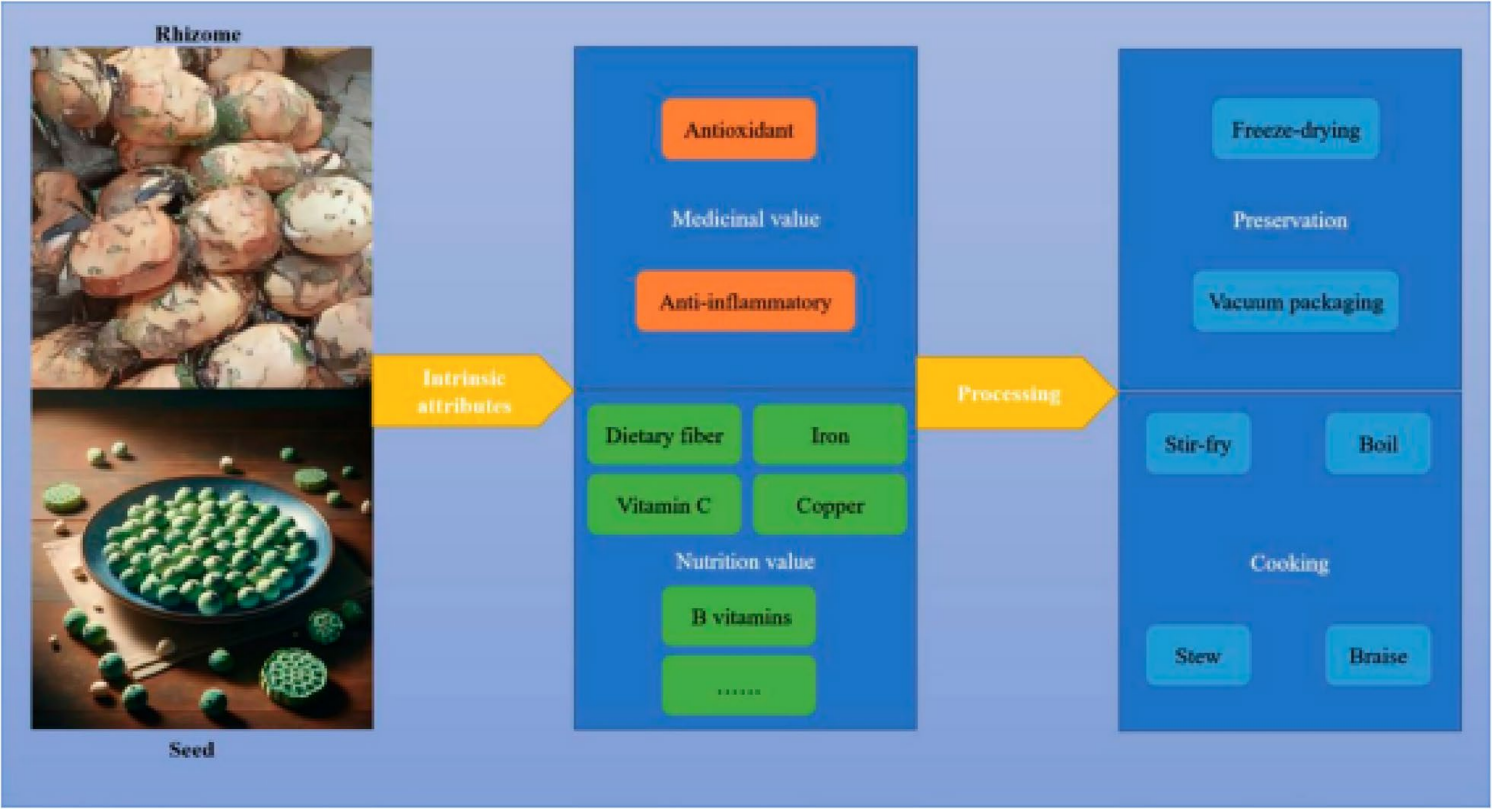


Corrected Fig. 3

**Fig. 3:**
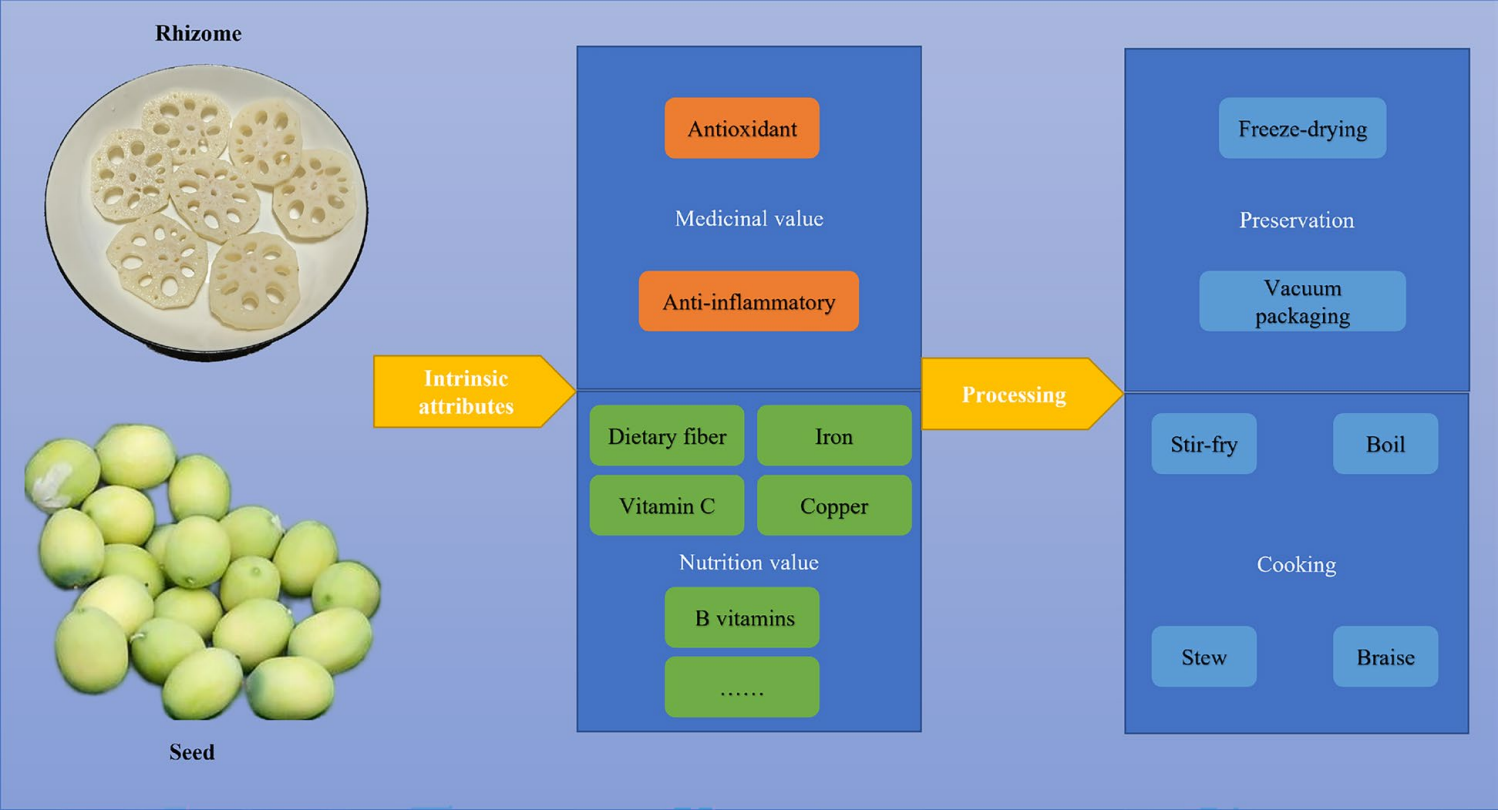
The schematic diagram of the nutritional and health benefits of lotus

Incorrect Fig. 4 
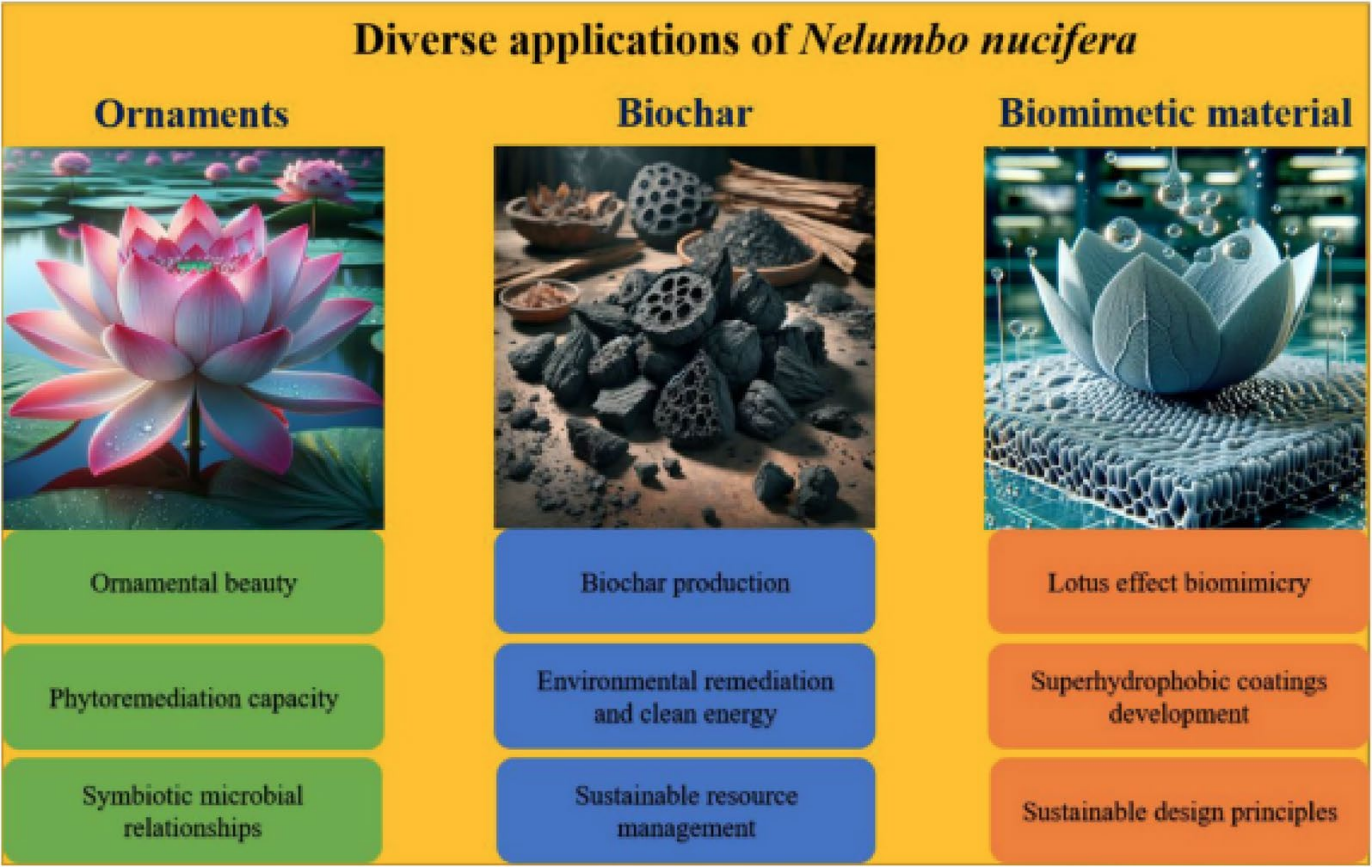


Corrected Fig. 4

**Fig. 4:**
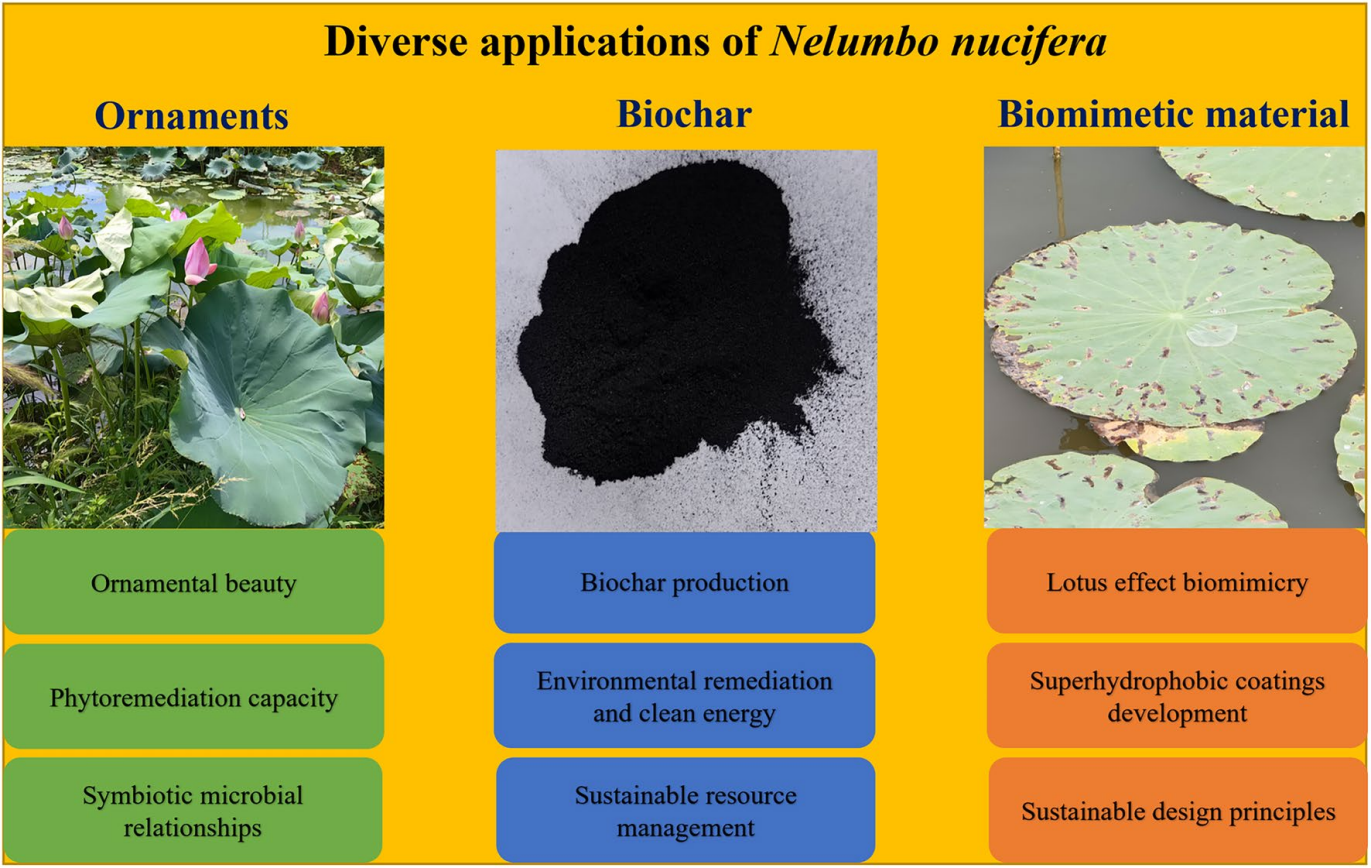
The schematic diagram of the diverse applications of lotus

Incorrect Fig. 5 
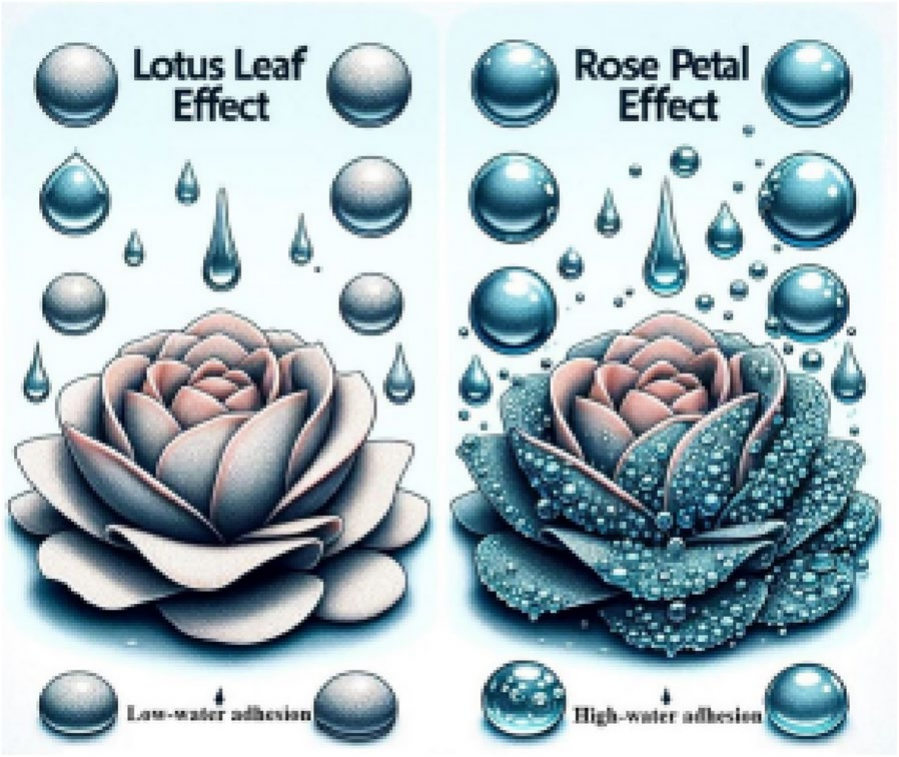


Corrected Fig. 5

**Fig. 5:**
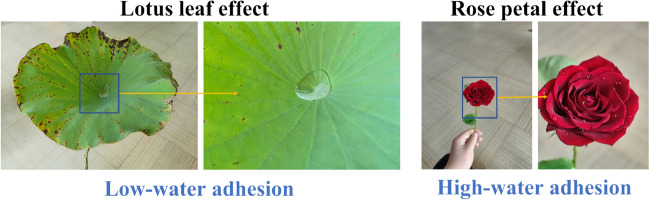
Illustration of rose petal and lotus leaf effects

Incorrect Table 1
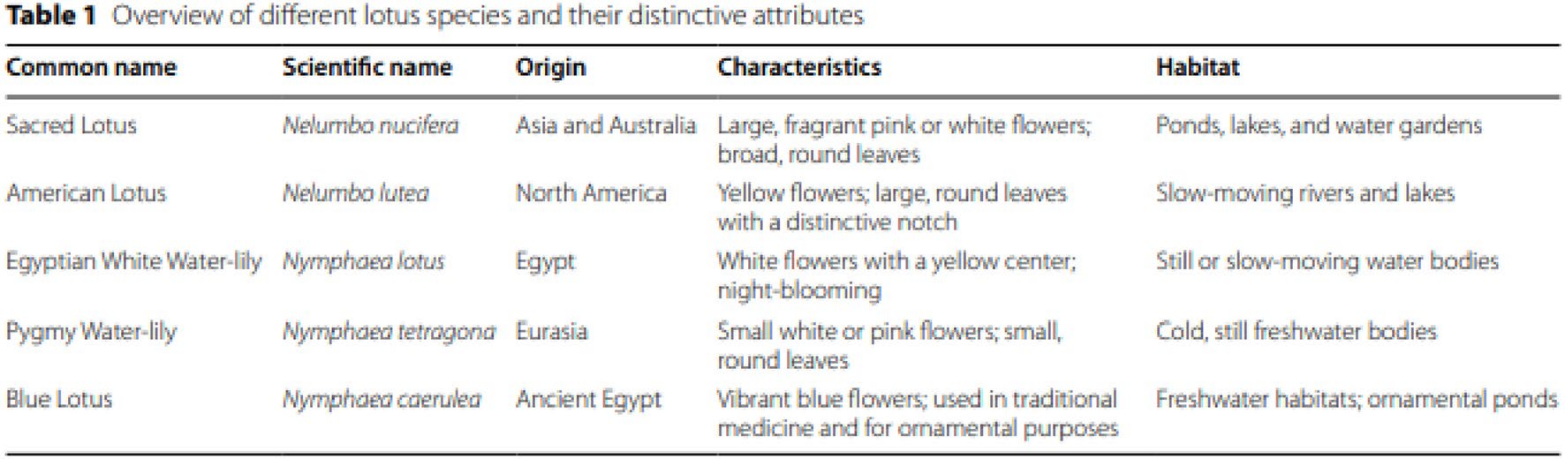


Corrected Table 1

**Table 1:** Overview of different lotus species and their distinctive attributes

Common name	Scientific name	Origin	Characteristics	Habitat
Sacred Lotus	*Nelumbo nucifera*	Asia and Australia	Large, fragrant pink or white flowers; broad, round leaves	Ponds, lakes, and water gardens
American Lotus	*Nelumbo lutea*	North America	Yellow flowers; large, round leaves with a distinctive notch	Slow-moving rivers and lakes
